# Dietary Strategies to Modulate Gut Microbiota in Metabolic Dysfunction-Associated Steatotic Liver Disease (MASLD)

**DOI:** 10.3390/nu17111906

**Published:** 2025-06-01

**Authors:** Federica Perazza, Laura Leoni, Beatrice Selvatici, Francesca Girolami, Alessia Bonalumi, Alice Beretta, Silvia Ferri, Maria Letizia Petroni, Fabio Piscaglia, Federico Ravaioli, Michele Sculati

**Affiliations:** 1 Department of Medical and Surgical Sciences, IRCCS Azienda Ospedaliero-Universitaria di Bologna, 40138 Bologna, Italy; federica.perazza@studio.unibo.it (F.P.); francesca.girolami@unibo.it (F.G.); fabio.piscaglia@unibo.it (F.P.); 2 Department of Dietetics and Clinical Nutrition, Maggiore-Bellaria Hospital, Azienda Unità Sanitaria Locale (AUSL), 40138 Bologna, Italy; laura.leoni@ausl.bologna.it; 3 Division of Internal Medicine, Hepatobiliary and Immunoallergic Diseases, IRCCS Azienda Ospedaliero-Universitaria di Bologna, 40138 Bologna, Italy; beatrice.selvatici@aosp.bo.it (B.S.); silvia.ferri@aosp.bo.it (S.F.); 4 HandyDiet SRL, 24128 Bergamo, Italy; alessia@handydiet.it (A.B.); alice@handydiet.it (A.B.); 5 Division of Endocrinology and Diabetes Prevention and Care, IRCCS Azienda Ospedaliero-Universitaria di Bologna, 40138 Bologna, Italy; marialetizia.petroni@unibo.it; 6 Department of Public Health, Experimental and Forensic Medicine, University of Pavia, 27100 Pavia, Italy; info@sculati.it

**Keywords:** microbiota, nutrition, metabolic dysfunction-associated steatotic liver disease, MASLD, metabolic dysfunction-associated steatohepatitis, MASH, liver, fibrosis, diet, portal hypertension, non-alcoholic fatty liver disease, NAFLD, non-alcoholic steatohepatitis, NASH, cirrhosis

## Abstract

Gut microbiota has become an area of increasing interest for its potential role in metabolic dysfunction-associated steatotic liver disease (MASLD) and its more advanced form, metabolic dysfunction-associated steatohepatitis (MASH)—now recognized as the most frequent liver disease worldwide. Research suggests that imbalances in the intestinal microbiota, including dysbiosis and increased intestinal permeability, may contribute to the pathogenesis of MASLD and progression to MASH. These changes affect insulin resistance and trigger inflammatory responses by disrupting the gut–liver axis. This review examined the current evidence connecting gut microbiota to MASLD and MASH, exploring how microbial shifts might influence liver health. Emerging strategies—such as probiotics, prebiotics, and targeted dietary changes—that may help prevent or manage these conditions are also discussed. Finally, key areas where further studies are required to understand the role of microbiota and its therapeutic potential are highlighted.

## 1. Introduction

Metabolic dysfunction-associated steatotic liver disease (MASLD) is currently the most common hepatic disease, affecting approximately 30–38% of the adult population [1,2,3]. In 2023, in a Delphi consensus, the term MASLD replaced the previous terminology, non-alcoholic fatty liver disease (NAFLD) and non-alcoholic steatohepatitis (NASH) [1]. In the new nomenclature, MASLD refers to a condition in which hepatic steatosis co-exists with at least one cardiovascular risk factor (high waist circumference, hypertension, impaired fasting glucose/glucose tolerance, hypertriglyceridemia, decreased levels of HDL cholesterol). The rationale behind the change in nomenclature is the need to abolish the terms “nonalcoholic” and “fatty” due to stigma. The terminology “steatotic liver disease” was approved because it included different etiologies of steatosis. Furthermore, a new category was added: metabolic and alcohol-related/associated liver disease (MetALD), in case of metabolic dysfunction-associated liver disease coexisting with significant alcohol consumption (140–350 g/week and 210–420 g/week for females and males, respectively) [1]. The growth in MASLD incidence and prevalence is related to the worldwide spread of obesity. However, in addition to the connection to obesity, MASLD presents a strong bond with type 2 diabetes (T2D), probably owing to the significant role of insulin resistance in the pathogenesis of both disorders [1].

Histologically, MASLD is characterized by abnormal fat storage in hepatocytes: steatosis is present in more than 5% of hepatocytes without significant ballooning, as other causes of liver disease are absent. MASLD may progress to metabolic-associated steatohepatitis (MASH), characterized by steatosis and inflammation with hepatocyte ballooning, an indicator of liver damage; the exacerbation of the disease includes hepatic fibrosis, cirrhosis, and hepatocellular carcinoma [4].

In March 2024, the Food and Drug Administration approved Resmetirom as the first drug for the treatment of MASH associated with fibrosis. However, since then, no pharmacological therapy for MASLD/MASH has been available. Lifestyle modification and weight loss >10% are the most effective strategies to reverse the disease [5]. Nevertheless, most patients with MASLD/MASH did not achieve such weight reduction [6]. Therefore, further research on other effective treatments is necessary.

Since altered gut microbiota (dysbiosis) plays a critical part in the pathogenesis of MASLD, it has started to gain attention as a possible target therapy for MASLD [7]. The gut–liver axis, a two-way connection involving the intestines, liver, and immune system, is disrupted in MASLD due to an imbalance in microbial composition. Dysbiosis results in intestinal permeability, allowing bacterial endotoxins to enter the portal circulation, provoking hepatic inflammation and insulin resistance. Additionally, altered microbiota can affect bile acid metabolism, short-chain fatty acid (SCFA) synthesis, and choline metabolism, all contributing to hepatic steatosis and fibrosis [8]. Alteration of gut microbiome has been connected not only with MASLD, but also with other metabolic dysfunctions like T2D and obesity [9,10,11]. Hence, modulation of the gut microbiota has been widely investigated as a target for treating the condition. Several therapeutic strategies for enhancing the heterogeneity and abundance of the gut microbiota have been investigated over the years, including probiotics, prebiotics, and tailored diet modifications. Understanding these microbial interactions offers potential therapeutic targets to mitigate MASLD progression, given that hepatocellular carcinoma may occur in a noncirrhotic liver [12].

This narrative review outlines the current evidence about dietary supplements that may modulate the microbiota and subsequently impact MASLD/MASH.

## 2. Data Sources and Searches

We conducted a literature search of English-language publications indexed in MEDLINE, the Cochrane Library, EMBASE, Web of Science, and PubMed, with coverage up to February 2025. The search strategy was tailored for narrative synthesis and employed combinations of the following keywords: non-alcoholic fatty liver disease, NAFLD, metabolic-associated steatotic liver disease, MASLD, non-alcoholic steatohepatitis, metabolic-associated steatohepatitis, MASH, liver disease, probiotics, prebiotics, microbiota, microbiome, gut, and gut–liver axis. Boolean operators (e.g., AND, OR) were used to combine terms where appropriate. No specific filters were applied for publication type or study design beyond language and relevance. The reference lists of relevant articles were also screened to identify additional sources. Inclusion criteria were (a) original research articles, clinical trials, meta-analyses, and narrative or systematic reviews; (b) studies directly addressing the role of microbiota, probiotics, or prebiotics in MASLD, NAFLD, MASH, or related hepatic conditions; (c) human and relevant preclinical studies. Exclusion criteria included (a) case reports, case series, brief communications, commentaries, editorials, and conference abstracts; (b) non-English language publications; (c) articles not directly relevant to the gut–liver axis or MASLD-related outcomes.

## 3. Gut Microbiota in MASLD/MASH

The ensemble of the organisms that inhabit the human body is called microbiota, whereas the term microbiome relates to the genomic constituent of microbiota [13]. It is composed of bacteria, fungi, archaea, protists, and viruses [14,15]. These micro-organisms lie not only in the gastrointestinal tract, but also in the epidermis, oral cavity, and respiratory and genitourinary tracts [16].

Gut microbiota is impacted by many aspects, such as early life microbiota composition; host DNA; and psychological, social, and geographic contexts. Diet is one of the major drivers of gut microbiota composition: in a real-world setting, thousands of different nutrients daily interact with trillions of microorganisms, in an intriguing multitude of possible pathways and interventions. In this field, probiotics are live microorganisms that, when dispensed in sufficient quantity, confer a potential benefit on the host [17]; conversely, prebiotics were described as a non-digestible food component that helpfully impacts the host by selectively stimulating the development and/or activity of one or a limited amount of bacteria and thus gives benefits to the host [18].

The intestinal microbiota has a fundamental role in metabolizing carbohydrates, proteins, polyphenols, vitamins, and bile [19,20]. Various processes by which the intestinal microbiota influences the worsening of MASLD/MASH have been described. Indeed, the increment of intestinal permeability, the translocation of dysbiotic microorganisms, and the synthesis of metabolites can be related to modifications in microbiota composition, and it can produce disordered inflammatory reactions that influence liver metabolism [21].

Studies have demonstrated that modifications in the quantity and quality of gut microbiota are connected to the development and progression of MASLD. Intriguingly, every stage of MASLD/MASH has a specific microbiota pattern [10], since the severity of the disease has been attributed to the depletion of commensal bacterial metabolic effects [11,22]. Actually, in MASLD, *Bacteroides* are reduced and *Firmicutes* and *Proteobacteria* are increased at the bacterial phylum grade [10]. Moreover, at the bacterial family grade, Enterobacteriaceae are enhanced, and, on the other hand, *Rikenellaceae* and *Ruminococcaceae* are reduced [10]. At the bacterial genera level, *Escherichia*, *Dorea*, and *Peptoniphilus* are demonstrated to be increased; and *Anaerosporobacter*, *Coprococcus*, *Eubacterium*, *Faecalibacterium*, and *Prevotella* are reduced [10]. Compared to MASLD, in MASH, the gut microbiome is characterized by a high representation of *Bacteroides*, whereas fibrosis presents elevated levels of *Ruminococcus* [11]. All of the above shows that in MASH, if compared with MASLD, gene expression implicated in lipopolysaccharide (LPS) synthesis in gut microbiota was increased [23]; this was recently confirmed by Barchetta et al. in a study in which LPS and its binding protein had been recognized as possible factors in the pathogenesis of MASLD [24]. Moreover, an increase in flagellar biosynthesis gene expression is linked with fibrosis [21]. Bacterial translocation, owing to enhanced gut permeability, and raised blood levels of LPS have been linked to MASLD [25].

The literature has so far demonstrated the critical role of gut microbiota in MASLD [10]. Strong evidence indicated that performing a fecal microbiome transplantation (FMT) on a germ-free murine model derived from a human with MASLD resulted in partial MASLD histological features [26]. Indeed, in 2023, Le Roy et al. highlighted that differences in microbiota composition may influence the answer to a high-fat diet (HFD) in mice [27]; such data confirmed the hypothesis that gut microbiota is implicated in the pathogenesis of MALSD, even independently of obesity [27]. Successively, Soderberg et al. performed FMT on germ-free mice from newborns of lean mothers and with obesity, respectively [28]. The mice with a transplanted fecal microbiome from mothers with obesity gave birth to mice that showed MASLD-like modifications, with periportal inflammation in addition to MASLD [28]. Moreover, experiments with FMTs from individuals with MASLD to germ-free murine models corroborated this pathogenetic thesis. Accordingly, Chiu et al. moved the gut microbiome from MASH subjects to germ-free murine models while feeding the mice with an HFD [29]. The authors showed that the mice increased fat weight, hepatic steatosis, inflammation, multifocal necrosis, and alanine aminotransferase (ALT) and aspartate aminotransferase (AST) levels, along with pro-inflammatory cytokines such as endotoxin and interleukin-6 (IL-6) [29]. Conversely, germ-free rats fed with an HFD showed lower lipid storage and liver inflammation. In 2018, Hoyles et al. also reported that microbiome transplantation from MALSD subjects to germ-free murine models led to hepatic steatosis and a MASLD gut microbiota mark [30].

## 4. Modulators of Gut Microbiota in the MASLD Pathogenesis

**Figure 1:**
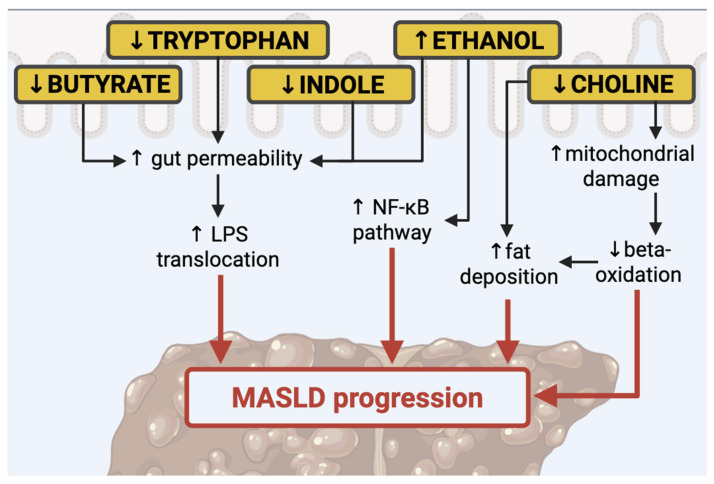
Principal modulators of gut microbiota in the MASLD. Nuclear factor-κB (NF-κB), lipopolysaccharide (LPS).

One of the most relevant types of prebiotics are indigestible carbohydrates, among them resistant starch, inulin, lignin, pectin, cellulose, fructo-oligosaccharides (FOS), and galacto-oligosaccharides (GOS). These nutrients reach the colon undigested, where they are degraded by carbohydrate-active enzymes (CAZymes), abundantly expressed by the microbiome. Those fibers can be fermented in the colon, producing SCFAs, such as acetate, propionate, formate, butyrate, lactate, and succinate. SCFAs are critical for maintaining enterocyte health and differentiation, mucus secretion, and preventing bacterial translocation [9,31] (see Figure 1).

### 4.1. SCFA

SCFAs are the results of the fermentation of nondigestible carbohydrates (NDC) that become available to the gut microbiota. The most important NDCs are acetate, propionate, and butyrate [32,33]. *Bacteroides* are the principal producers of propionate and acetate, and conversely, *Firmicutes* of butyrate [32]. Butyrate and propionate are reported to be remedies for bowel inflammation [34]. In rats, acetate and propionate supplementation reduced lipogenesis and fat storage, protecting them from HFD-induced weight gain [35].

Butyrate is an efficient anti-inflammatory mediator [36,37]. Since butyrate decreases the inflammation in the gut, studies have demonstrated that it may discourage the progression of inflammatory processes systemically. Indeed, butyrate can stimulate the activation of the regulatory T cells, which seem to be responsible for the inactivation of T-helper 17 (Th-17) cells and T cells; this results in the extinguishment of pro-inflammatory pathways [36,37]. Rationally, low levels of butyrate are responsible for low-grade inflammation and a less effective anti-inflammatory response. Butyrate is connected to the reduction in inflammatory pathways through different actions. Firstly, through the improvement in tight junction function. This effect is displayed by the induction of mucin production, which preserves the integrity of the intestinal barrier and prevents the translocation of bacteria and their products into the portal circulation. Secondly, through the inhibition of histone deacetylases. Lastly, it serves as an energy provider for colonocytes to maintain gut health [38,39,40].

In MASLD, reduced levels of butyrate may result in increased gut permeability, which is connected to the risk of LPS transfer in the bloodstream. LPS is engaged in the development of MASLD, as Fei et al. demonstrated, showing that endotoxin-producing bacteria were over-presented in people living with obesity [41]. In a paper published in 2020, it has been highlighted that LPS levels in the blood and liver are higher in individuals with MASLD than in healthy subjects [42].

On the other hand, butyrate may stimulate the expression of glucagon-like peptide-1 receptor (GLP-1r) to moderate the severity of MASLD [23]. In a paper, Svegliati-Baroni et al. showed that the expression of the GLP-1r is decreased in the hepatocytes of murine models fed HFD and patients with MASH [43]. Moreover, it was noticed that the activation of GLP-1r in the hepatocytes increased the oxidation of β-fatty acids and ameliorated insulin resistance [43]. Zhou et al. demonstrated that GLP-1 levels are equal in patients with MASLD and subjects without MASLD, but in people living with MASLD, the GLP-1r is low-expressed [23]. Intriguingly, in a rat fed a high-fat diet, supplementation with butyrate is connected to an enhanced expression of GLP-1r, which alleviates liver steatosis [23]. Moreover, in a recent paper, patients with MASLD had higher plasma levels of propionate, formate, valerate, and α-methylbutyrate but reduced acetate concentrations [44]. Consistently, in subjects with MASLD, marked fibrosis was positively connected with propionate, butyrate, valerate, and α-methylbutyrate [44]. On the other hand, in another study, it has been reported that butyrate supplementation prevents the progression from liver steatosis to steatohepatitis due to the protection from the induction of inducible nitric oxide synthase and lipid peroxidation in the liver [45]. In a paper published in *Nature*, authors showed that acetate is strongly connected with hepatic de novo lipogenesis, in particular, as a result of the microbial metabolism of fructose [46].

While SCFAs—particularly butyrate—play key roles in gut and liver homeostasis, conflicting evidence about their association with both protective and fibrogenic pathways in MASLD underscores the need for further mechanistic clarification.

### 4.2. Polyphenols

Polyphenols are a varied class of organic substances with prebiotic effects [47]. In nature, there are more than 8000 different compounds that, according to their chemical structure, can be categorized into flavonoids, lignans, phenolic acids, stilbenes, non-phenolic metabolites, and other polyphenols [48,49]. Like indigestible carbohydrates, most dietary polyphenols are not absorbed in the small intestine. When they arrive in the large bowel, they perform health benefits directly, stimulating the growth of advantageous microorganisms and inhibiting the spread of dangerous ones, and indirectly, by their metabolites, such as SCFAs [50]. Among polyphenols, flavonoids, particularly anthocyanins, have been shown to improve metabolic disorders such as MASLD. They increase the abundance of beneficial gut bacteria, including *F. prausnitzii*, *Lactobacillus*, and *E. rectum*, while reducing pathogens such as *Desulfovibrio* and *Enterococcus* [50,51]. Other polyphenols with a specific effect on the improvement of MASLD are stilbenes: 2,3,5,4′-tetrahydroxy-stilbene-2-O-β-d-glucoside has been shown to improve liver mitochondrial dysfunction and lipid accumulation [52]. Another group of pigmented biologically active phytochemicals is betacyanins. They are studied for their ability to display beneficial actions in animal models of MASLD through modulation of the intestinal microbiota, reducing the abundance of *Firmicutes* and increasing that of *Bacteroides* at the phylum level. Furthermore, betacyanins also dramatically increase the amount of *Akkermansia* at the genus level [53].

### 4.3. Bile Acids

Bile acids (BAs) are molecules synthesized in the liver from cholesterol and stored in the gallbladder. Besides helping the absorption of lipids, bile acids are also crucial in the metabolism of glucose. The intestinal microbiota transforms primary bile acids, cholic acid (CA), and chenodeoxycholic acid (CDCA) into secondary bile acids like deoxycholic acid (DCA), lithocholic acid (LCA), and ursodeoxycholic acid (UDCA) [54,55]; this process occurs in the distal small intestine and large bowel. BA is indirectly engaged in antimicrobial defenses operated by the farnesoid X receptor (FXR). The activation of FXR decreases fatty acid and triglyceride production in the liver by reducing the expression of the liver X receptor (LXR) and Sterol Regulatory Element Binding Protein 1C (SREBP-1C) [56]. Accordingly, FXR-deficient mice show decreased insulin sensitivity and reduced glucose tolerance [57]. On the other hand, FXR activation by specific agonists suppresses bile acid and fatty acid synthesis and increases glucose and insulin sensitivity in murine models with obesity and diabetes. Furthermore, FXR stimulation seems to reduce primary biliary cirrhosis and MASH by diminishing the bile acid pool and liver fibrosis [58,59]. In a meta-analysis including 19 studies and 154,807 individuals, it emerged that total BA levels in patients with MASLD were more elevated than those in individuals without MASLD; in particular, UDCA, taurococholic acid (TCA), CDCA, taurochenodeoxycholic acid, and glycocholic acids were elevated. Remarkably, TCA, taurodeoxycholic acid, taurolithocholic acids, and glycolithocholic acids demonstrated a possible ability to discriminate MASH [60].

### 4.4. Tryptophan

Tryptophan is an essential amino acid, which means that it needs to be integrated with the diet to be available for the organism; this aromatic amino acid disposes of various pathways to be metabolized. When tryptophan is decomposed by tryptophan hydroxylase 1 and 2 (Thp1/Thp2), serotonin (5-HT) becomes available; moreover, in the indole pathway, tryptophan is transformed into indole, plus other metabolites [61].

Existing data have shown that reduced levels of tryptophan are connected to alteration of the intestinal barrier integrity [62,63]; consequently, it has been demonstrated that supplementation with tryptophan increases intestinal barrier integrity [64]. The positive effect of this amino acid on gut health leads to improvement in liver steatosis. Consequently, MASLD is characterized by poor levels of tryptophan, since in this condition, its metabolism is impaired [65]. In a paper by Ritze et al., the authors found that tryptophan supplementation improved liver steatosis induced by a fructose-based diet in rats [65].

Moreover, the indole metabolite is crucial in preserving intestinal wall integrity: in selected mice, depletion of indole was associated with impaired intestinal integrity [64]. Accordingly, supplementation with indole metabolite determines an increase in the expression of molecules implicated in the assembly of tight junctions. Furthermore, another study highlighted that indole levels in the bloodstream were inversely associated with obesity and liver steatosis [66]. In line, Ji et al. reported that indole-3-acetic, derived from indole, reduces lipogenesis and inflammation in the liver of mice nourished with HFD [67].

Finally, serotonin has also been connected with MASLD since high levels of serotonin are accountable for inhibiting the energy expenditure of brown adipose tissue [68]; this evidence is confirmed by Arto et al., who demonstrated in a cohort of 76 patients that alteration in tryptophan catabolism is related to MASLD, highlighting the potential utility of targeting these pathways in therapeutic approaches [69].

### 4.5. Choline and Trimethylamine

Choline is a fundamental component of cellular and mitochondrial membranes and serves as a precursor of acetylcholine, a key neurotransmitter. Among its derivatives, phosphatidylcholine plays a central role in the formation of very low-density lipoprotein (VLDL) from triglycerides and in the solubilization of bile acids for their elimination [70,71]. Reduced choline levels alter mitochondrial membrane composition, compromising their function and contributing to impaired adenosine triphosphate (ATP) synthesis and reduced beta-oxidation. This metabolic dysfunction promotes the progression of hepatic steatosis [72,73]. Accordingly, diets deficient in choline are commonly used in animal models to investigate the pathogenesis and progression of MASLD, as they replicate features observed in humans, such as hepatic triglyceride accumulation [74]. For instance, Arao et al. demonstrated that a choline/methionine-deficient diet in a murine model induced MASH and was associated with reduced mitochondrial DNA content [75]. Choline availability is also influenced by the gut microbiota. Choline is metabolized by intestinal microbes, which can reduce its systemic bioavailability and increase the risk of deficiency [76]. Furthermore, the microbiota facilitates the conversion of choline into trimethylamine (TMA), which is absorbed and converted by the liver into trimethylamine N-oxide (TMAO [77]. Excessive TMAO production contributes to further reductions in circulating choline levels, interfering with hepatic VLDL export and bile acid synthesis. These alterations may worsen hepatic fat accumulation, oxidative and inflammatory damage, and disrupt glucose metabolism [78,79]. Consistent with these mechanistic insights, a recent randomized controlled trial by Perva et al. showed that nutritional supplementation containing choline, along with other compounds (5-MTHF, betaine, alpha-linolenic acid, eicosapentaenoic acid, choline bitartrate, docosahexaenoic acid, and vitamin B12), had beneficial effects on liver fibrosis and steatosis parameters in individuals with metabolic syndrome and obesity [80]. However, the role of TMAO in MASLD remains controversial. In a recent study, TMAO was associated with hepatic lipid accumulation and accelerated disease progression in murine models [81]. Additionally, TMAO has been shown to impair intestinal barrier integrity at multiple levels, promoting liver endothelial dysfunction, capillarization of liver sinusoidal endothelial cells (LSECs), and alterations in macrophage polarization [81]. A systematic review and meta-analysis published in 2022 further supported TMAO’s involvement in hepatic triglyceride accumulation, impaired cholesterol transport, disrupted glucose and energy homeostasis, and alterations in bile acid metabolism [82].

Nonetheless, despite growing preclinical evidence, further research is required to clarify the causative role of TMAO in human MASLD and to determine whether it represents a therapeutic target or a secondary marker of disease.

### 4.6. Ethanol

Ethanol is a derivative of saccharolytic fermentation. In a paper published in 2000, researchers suggested a relationship between ethanol blood concentrations and changes in intestinal microbiota [83]. Additionally, research indicates that dysbiosis in patients with MASH is linked to ethanol-producing microorganisms, including *Escherichia coli*, *Bacteroides*, *Bifidobacterium*, and *Clostridium* [84,85]. In particular, one study pointed out an increase in ethanol levels in subjects with MASH compared to fit individuals or patients living with obesity but without MASH [84]. Ethanol produced by gut bacteria, along with its oxidized compound, acetaldehyde, may contribute to the worsening of MASLD This occurs through direct toxic action on liver cells, impairment of the intestinal barrier, elevated portal endotoxemia, and activation of the nuclear factor-κB (NF-κB) pathway, which heightens inflammatory pathways in peripheral areas’ cells [86,87].

## 5. Drugs and Microbiota Modulation

### 5.1. Metformin

Metformin is the first-line glucose-lowering therapy in type 2 diabetes mellitus (DM2), although its mechanism of action remains complex and not fully understood [88]. Recent studies have shown that metformin treatment is associated with notable changes in gut microbiota composition, including an increase in the abundance of *Escherichia coli* and a reduction in *Intestinibacter* [89,90]. Additionally, metformin influences the abundance of bacteria involved in the production of short-chain fatty acids (SCFAs), particularly enhancing the presence of *Akkermansia muciniphila* [89,90]. *Akkermansia muciniphila* plays a key role in maintaining intestinal barrier integrity and promoting SCFA production. These effects, in turn, exert beneficial actions on adipose tissue, skeletal muscle, and the liver by improving insulin sensitivity [91].

Supporting the relevance of microbiota in mediating the effects of metformin, a study demonstrated that transplantation of gut microbiota from metformin-treated individuals into germ-free mice led to improved blood glucose levels. This finding suggests that the glycemic benefits of metformin may be partially mediated through alterations in gut microbial composition [90].

Consistently, evidence from three meta-analyses indicates that metformin exerts beneficial effects on MASLD, including reductions in AST and ALT levels and decreased liver fat content [92,93,94]. However, these findings are not universally confirmed. Other studies, including a meta-analysis, reported neutral effects of metformin on hepatic fat accumulation and transaminase levels [95,96,97,98,99]. These discrepancies may be attributed to heterogeneity in study design, treatment duration, or patient populations.

Overall, while the interaction between metformin and the gut microbiota appears to contribute to its metabolic effects, further research is needed to clarify the extent and consistency of its potential in the management of MASLD.

### 5.2. Rifaximin

Rifaximin is an oral, non-systemic antibiotic that exerts its action locally in the gut without entering the bloodstream (it is not absorbed). It displays broad-spectrum antimicrobial activity against both aerobic and anaerobic and Gram-positive and Gram-negative bacteria, many of which are involved in the production of LPS in the intestinal lumen [100,101]. Rifaximin is widely used in clinical practice for the management of hepatic encephalopathy, traveler’s diarrhea, and irritable bowel syndrome [102,103,104]. Beyond its established antimicrobial applications, recent studies have explored its potential role in liver-related diseases, including MASLD and MASH. In a recently published RCT, the combination of rifaximin with a probiotic and prebiotic—administered alongside metformin—in patients with MASLD and T2D led to the resolution of subclinical inflammation and improved intestinal barrier integrity [105]. Notably, the intervention group also showed reductions in AST and ALT levels, as well as an improvement in the degree of liver steatosis [105]. Supporting these findings, a previous study by Gangarapu and colleagues reported that short-term rifaximin treatment in patients with MASH resulted in decreased LPS production, which was associated with lower levels of transaminases, ferritin, and LDL cholesterol [102]. However, these beneficial effects were not observed in patients with MASLD in the same study, suggesting that the therapeutic response to rifaximin may differ depending on disease stage or phenotype [102].

In another RCT, rifaximin therapy led to reductions in endotoxin levels, cytokeratin-18, transaminases, insulin resistance, and MASLD-liver fat score, with overall improvement in MASH parameters [106].

Taken together, the available evidence suggests that rifaximin may have a beneficial impact on MASH by targeting the inflammatory component of the disease, although further studies are needed to clarify its role across the MASLD spectrum.

## 6. Diet and Gut Microbiota Modulation

### 6.1. Fibers

Different studies have evaluated the impact of specific fibers on the intestinal microbiota: fermentable dietary fibers, such as inulin, oligofructose, FOS, or GOS, have a beneficial action on microbiota composition and derived metabolites, with individual differences in the expected increase of SCFAs production [107].

RCTs have shown that fiber-rich whole grains significantly reduce lipopolysaccharide binding protein (LBP) and inflammation, improving gut barrier function and microbiota diversity [31]. On the contrary, low-fiber diets increase mucus-degrading microorganisms (*Akkermansia muciniphila* and *Bacteroides caccae*) and decrease fiber-degrading species (*Bacteroides ovatus* and *Eubacterium rectale*), reducing SCFA production [108]. Since the mucus layer in the colon acts as a primary defense against pathogens, the weakening of this barrier has a significant impact on susceptibility to gastrointestinal infections [109].

Within different fermentable nutrients, resistant starch (RS) reaches the large intestine in far more significant amounts than other substrates, including non-starch polysaccharides, oligosaccharides, unabsorbed sugars, and dietary protein. This considerable quantity accessible for fermentation by intestinal microbiota leads to a corresponding production of SCFAs [110] and to benefits related to their production. RS is a prebiotic fiber that can be categorized into five categories. RS1 is physically unreachable because it is confined within cell walls or in whole or partially milled grains seeds; RS2 is finely textured native starch characterized by high crystallinity structure; RS3 is subjected to retrograde metamorphism or recrystallization starch; RS4 is starch that has been chemically modified or mixed with non-starch components bonds; and RS5 serves as a starch-lipid complex primarily associated with amylose–lipid complexes [111,112].

Concerning liver metabolism, RS supplementation (40 g/die) led to a 5.89% reduction in hepatic lipid content in a 4-month randomized placebo-controlled clinical trial in subjects with MASLD [113]. Moreover, RS reduced the presence of *Bacteroides stercoris*, which correlates with MASLD progression and improved liver inflammation and steatosis [113]. Omnivores may experience gut microbial signatures with other diet patterns, as long as they include a comparable variety of fiber-rich plant-based foods in their diets [114].

### 6.2. Lipids

Dietary lipids quality and quantity have beneficial or detrimental health effects, some of which are mediated by induced changes in the gut microbiome. An HFD induces an overrepresentation of LPS-expressing bacteria, increasing the risk of rising LPS circulating levels [115,116]. This condition, called “metabolic endotoxemia”, is associated with enhanced intestinal permeability, probably as a result of reduced tight junction protein expression. These negative effects are mainly caused by the overconsumption of saturated fats, while the consumption of unsaturated fats appears to be protective [117]. A diet rich in unsaturated fats produces an increase in the presence of beneficial taxa such as *Akkermansia* and *Bifidobacterium*, and a decrease in critical microorganisms such as *Streptococcus* and *Escherichia* [118]. Saturated fatty-acid-rich foods, for example, butter or lard, have been reported to augment the presence of *Lachnospiraceae Blautia*, *Roseburia*, *Lachnospira*, *Agathobacter*, *Fusicatenibacter*, *Lachnoclostridium*, *Bacteroides*, *Turicibacter*, and *Bilophila* spp., which promote inflammation [117,119]. An adequate intake of omega-3 polyunsaturated fatty acids (PUFAs) is connected with increased quantities of *Bifidobacterium* and *Lactobacillus*, as well as a remarkable increase in several SCFA (butyrate)-producing genera, including *Blautia*, *Bacteroides*, *Roseburia*, and *Coprococcus* [120]. This evidence was also demonstrated in a randomized intervention trial, where an omega-3 fatty acid supplementation exhibited a potential prebiotic effect, modifying the gut microbiome composition, leading to substantial increases in iso-butyrate and isovalerate [121]. Finally, omega-3 PUFA supplementation is related to a reversible enhancement in various SCFA-producing bacteria, no matter the route of administration. However, a connection between the gut microbiome and systemic omega-3 PUFA exposure has not yet been demonstrated [120].

### 6.3. Proteins

Only a few studies examined the impact of overall dietary protein intake on the intestinal microbiota in humans living with overweight or obesity. It is noteworthy that consumption of a high-protein diet (25 percent of energy intake) from various sources (meat, fish, dairy and plant) showed no effect on alpha or beta diversity, but when corrected for fat intake, a change in gut microbiota could be reported, largely due to the amount of protein consumed [122]. Specific amino acids may influence the gut microbiota composition, either directly or through their metabolites. L-carnitine is metabolized by the intestinal microbiota to trimethylamine (TMA), which in turn is transported from the portal circulation to the liver and converted to trimethylamine N-oxide (TMAO); the latter is a metabolite correlated with atherosclerotic cardiovascular disease and associated with the risk of MASLD [123]. In rats and humans, specific microorganisms of the gut microbiota have been linked with the potential to convert L-carnitine to TMA, with a common association with *Prevotella* [31].

### 6.4. Food Additives

The food industry has been increasingly utilizing food additives to enhance food and beverages’ preservation, freshness, taste, texture, or appearance. Studies in mice have illustrated that common dietary emulsifiers could modify intestinal microbiota diversity and cause gut microbiota dysbiosis. These modifications lead to bacterial incursion into the inner mucus layer and enhance inflammation, raising circulating LPS [124,125]. Low doses of two common emulsifiers, carboxymethyl cellulose and polysorbate-80, when tested in cultures with human intestinal microbiota, induced elevated levels of bioactive flagellin, likely due to dysbiosis or changes in bacterial gene expression [124]. A RCT aimed to explore these effects in humans through a short-term dietary intervention revealed that dietary emulsifiers reduced microbiota diversity and SCFA synthesis [126]. Non-caloric artificial sweeteners (NAS) are another extensively (https://synonyms.reverso.net/sinonimi/en/extensively, accessed on 27 January 2025) consumed group of food additives; some studies have linked NAS consumption to dysbiosis, but other data show heterogeneous results, and more research on humans is needed [127,128]. Besides artificial sweeteners, food additives such as emulsifiers, preservatives, flavor enhancers, and dyes can compromise the integrity of the intestinal wall and alter the structure of gut microbiota, which may trigger inflammation. These processes can result in oxidative stress that the body may not adequately counter, increasing the risk of impaired lipid metabolism in the liver [129] 

Ultra-processed foods (UPFs) are industrially made and are primarily characterized by high levels of added sugars, saturated fats, salt, and additives, while lacking essential nutrients and fiber [130]. Because of their high calorie density and poor nutrient quality, UPFs lead to a higher overall caloric consumption, resulting in weight gain and obesity [131]. Furthermore, due to their relationship with insulin resistance, UPFs are central in the pathogenesis of MASLD. They typically have high fructose levels, metabolized in the liver, and are potentially responsible for increased fat storage [132]. The harmful impact of fructose contained in UPFs is intensified by ingredients like saturated fats, food additives, and the lack of fiber and vital nutrients, all of which promote de novo lipogenesis and worsen metabolic dysfunction [133]. Additionally, UPFs can trigger oxidative stress and inflammation, crucial steps in advancing MASLD to more severe forms such as steatohepatitis [134]. Moreover, the low nutritional quality of UPFs—characterized by a lack of fiber and antioxidants and an excess of additives—can disturb the gut microbiome, resulting in adverse health consequences [135].

The overall composition of a diet interacts with gut microbiota, affecting both the types and quantities of bacteria, as well as their growth and interactions with enterocytes. Therefore, employing specially structured diets could be an effective intervention for MASLD.

## 7. Diet Manipulation for Enriching Gut Microbiota in MASLD

Several foods can deliver different pre- and probiotics to the gastrointestinal tract, and the overall diet may have an effect on the composition of the microbiota. Hence, a growing body of evidence shows that dietary modulation can have a therapeutic effect on MASLD.

### 7.1. Probiotics

In a RCT published in 2017, Manzhalii et al. illustrated that in a cohort of subjects with MASLD, the group that received not only the prescription of a low-calorie/low-fat diet but also a mixture of probiotics (*L. casei*, *L. rhamnosus*, *L. bulgaris*, *B. longum*, and *S. thermophilus*) experienced an improvement in liver inflammation [136]. This evidence was anticipated in 2012 by Malaguarnera et al., [112] who recruited for a RCT of 66 patients with biopsy-proven MASH at baseline. After 24 weeks, the intervention group treated with *Bifidobacterium Longum* plus FOS plus lifestyle modifications experienced a reduction in TNF-α, CRP, AST levels, homeostatic model assessment for insulin resistance (HOMA-IR), serum endotoxin, steatosis, and the MASH activity index [137]. Furthermore, in a cohort of subjects with MASLD, Sepideh et al. [113] observed that supplementation with probiotics (*Lactobacillus casei*, *Lactobacillus acidophilus*, *Lactobacillus rhamnosus*, *Lactobacillus bulgaricus*, *Bifidobacterium breve*, *Bifidobacterium longum*, and *Streptococcus thermophilus*) for 8 weeks improved insulin resistance, TNF-α, and IL-6 decreased significantly compared to baseline [138] Adversely, another RCT evaluated the impact of the co-administration of probiotics and prebiotics (FOS plus *Bifidobacterium animalis* subspecies lactis BB-12) vs. placebo in patients with MASLD; data showed that after one year of supplementation with a symbiotic cocktail (probiotic and prebiotic), the fecal microbiome varied but did not ameliorate liver steatosis or markers of fibrosis [139].

### 7.2. Sulforaphane

In a RCT with 140 participants with MASLD enrolled, the intervention group, which took six broccoli seed tablets rich in sulforaphane—an isothiocyanate present in cruciferous—after 12 weeks of supplementation, showed an increase in GLP-1. On the other side, levels of glucose, insulin, and HOMA-IR were reduced [140].

### 7.3. Polyphenol

In a RCT, Agrinier et al. showed that a polyphenol-rich extract from the Amazonian berry camu-camu may reduce liver steatosis in adults with overweight, after 12 weeks of treatment [51]. Moreover, in a pilot paper, authors hypothesized that onion polyphenols could potentially be utilized to compose dietary supplements as potential multi-target-directed ligands in MASLD [141]. Finally, a scoping review recently published—aiming to organize the present data on bioactive-substance-based supplementation for individuals with MASLD—showed that curcumin, silymarin, resveratrol, coffee, green tea, and berberine were the most studied bioactive substances [142]. However, data are abundant just for curcumin and silymarin [142].

### 7.4. Inulin

Back in 2019, researchers found that inulin-propionate ester, projected to selectively carry propionate to the colon, inulin, and cellulose were effective in improving insulin sensitivity for twelve adults with overweight or obesity but without diabetes, after 42 days of supplementation [143]. In a small RCT, Reshef et al. [144]. evaluated 8 individuals with MASLD and metabolic syndrome who received inulin-type fructans compared with 11 who received a placebo. There were no significant changes in liver steatosis, liver function markers, and inflammatory panel (fibroblast growth factor-19 and LPS) from baseline to the end of the treatment in both the prebiotic and placebo groups [144].

In 2020, Lin Chong demonstrated in a RCT that a short-term treatment with metronidazole, followed by a probiotic inulin supplementation, may reduce ALT levels in patients with MASLD more effectively than a short-term very low-calorie diet (VLCD) [145]. The authors hypothesized that the positive metabolic effects of short-term VLCD in adults with MASLD could be accentuated by metronidazole to treat dysbiotic gut microbiota, followed by a period of inulin supplementation as a maintenance [145].

### 7.5. Diet

**Figure 2:**
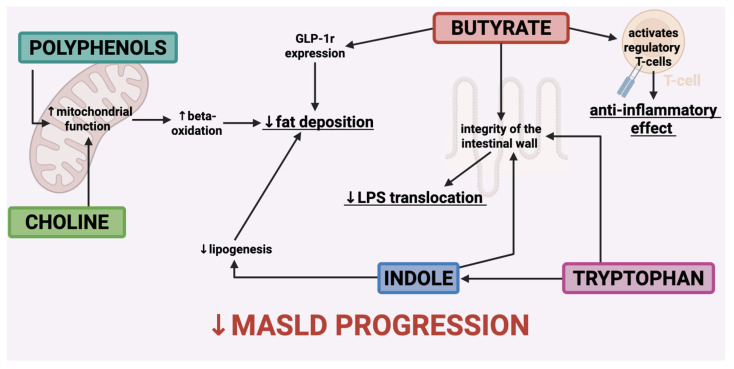
The primary metabolites that contribute to the alteration of the microbiota exert a positive influence on the natural progression of MASLD. Receptor of Glucagon-Like Peptide—1 (GLP-1 r); lipopolysaccharide (LPS).

As expected, in a RCT published in 2022, the authors pointed out that a low glycemic index Mediterranean diet plus physical activity determines improvement in liver steatosis measured through controlled attenuation parameter (CAP, Fibroscan^TM^, Echosens, Paris, France), an increase in the *Firmicutes phylum* and *Ruminococcaceae*, bacteria linked to liver protection [146]. On the other side, Jian et al. [122] evaluated the impact of an excess of 1000 kcal/day of diets rich in either saturated fat, unsaturated fat, or simple sugars for 3 weeks; the researchers reported that high intake of saturated fat enhanced *Proteobacteria* concentrations and sugar overfeeding increased *Lactococcus* and *E. coli*. On the other hand, unsaturated fat increased butyrate producers. In this study, the carriage of *Bilophila* was recognized as a possible risk factor for diet-determined hepatic steatosis in humans [147]. Moreover, in a RCT that enrolled 34 subjects with MASLD, it was shown that a freshwater fish-based diet may be beneficial to MASLD by regulating intestinal microbiota and its metabolites [148]; indeed, data highlighted that *Faecalibacterium* enhanced under the freshwater fish-based diet was positively associated with the levels of propionic acid, butyric acid, valeric acid, but negatively with acetic acid, isobutyric acid, isovaleric acid, and hexanoic acid levels [148]. Finally, Chen et al. reported that daily intake of yogurt was superior to milk in improving insulin resistance and liver fat in Chinese women living with obesity, MASLD, and metabolic syndrome [149]. Probiotic yogurt and its consumption are inversely associated with the prevalence of MASLD [150]. In a RCT that investigated the effects of symbiotic yogurt, with prebiotics (inulin) and probiotics (*Bifidobacterium animalis*), a significant reduction in hepatic steatosis and liver enzymes was observed in the intervention group [151]. Another double-blind RCT investigated the effects of probiotic yogurt consumption in 72 patients affected by MASLD. The study showed a reduction of 4.67 and 5.42% in concentrations of ALT and AST, respectively, compared with the control group [152]. A cross-sectional study, conducted among adults in the 2011–2016 National Health and Nutrition Examination Survey, linked the intake of probiotic yogurt with a beneficial action on liver steatosis [53] (see Figure 2).

## 8. Conclusions

This narrative review examines the impact of microbiota on the pathogenesis of MASLD and the potential for microbiota manipulation to enhance metabolic liver disease management. Dysbiosis is highlighted as a significant factor contributing to enhanced intestinal permeability and systemic inflammation, which is why research has focused on various therapeutic strategies, such as the administration of probiotics, prebiotics, symbiotics, and specific dietary patterns. Promising results from preclinical studies indicate that targeted supplementation could impact the progression of MASLD by restoring microbial balance. However, further standardized studies involving humans are essential to clarify the microbiota’s effective role as a therapeutic target for metabolic liver disease (see Table 1).

## Figures and Tables

**Table 1:** Most relevant studies concerning microbiota manipulation through diet in humans. Randomized controlled trial (RCT); metabolic-associated steatohepatitis (MASH); body mass index (BMI); metabolic-associated steatotic liver disease (MASLD); alanine transaminase (ALT); aspartate transaminase (AST); fructo-oligosaccharides (FOS); low-density lipoprotein (LDL); C-reactive protein (CRP); homeostatic model for insulin-resistance (HOMA-IR); tumor necrosis factor α (TNF)-α; interleukin-6 (IL-6); lipopolysaccharides (LPS); glucagon-like peptide 1 (GLP-1); inulin-type fructans (ITFs); very-low-calorie diet (VLCD); controlled attenuation parameter (CAP); gamma glutamyl transferase (GGT); insulin resistance (IR).

Treatment	Author	Study Design	Cohorts	Dosage	Effects	Notes
Low-fat/low-calorie diet + probiotics cocktail	Manzhalii et al. [136]	RCT	75 MASH	0–90 g fat/day and 1800 kcal/day + probiotics once daily for 12 weeks	- Reduced BMI - Reduced cholesterol, ALT, AST - Improved liver stiffness - Increase in Bifidobacteria and Lactobacilli and normalization of other bacterial species	No significant change was registered for pathogenic enterobacteria
Bifidobacterium longum with FOS	Malaguarnera et al. [137]	RCT	66 MASH—biopsy proven	*Bifidobacterium longum* with FOS + lifestyle modifications vs. lifestyle modifications for 24 weeks	- Reduced ALT, LDL, CRP, HOMA-IR, MASH activity index, TNF-α, endotoxin - Reduced steatosis	Biopsy at the beginning and the end of the follow-up (24 weeks)
Multistrain probiotics	Sepideh et al. [138]	RCT	42 MASLD	Probiotics vs. placebo for 8 weeks	- Reduced insulin, fasting blood glucose and insulin resistance - Reduced IL-6 and TNF-α	
Symbiotics (probiotics + prebiotics)	Scorletti et. al. [139]	RCT	104 MASLD	FOS + probiotics for 10–14 months	- Modification of the fecal microbiome - No improvement of liver fat content or markers of liver fibrosis	
Sulforaphan	Tian et al. [140]	RCT	36 participants with MASLD	Six broccoli seed tablets (rich in Sulforaphane 42 mg d^−1^) vs. placebo for 12 weeks	- Reduced blood glucose and HOMA-IR - Enhanced in Bacteroidaceae, Lactobacillaceae and Bifidobacteriaceae - Increased tight junctions - Reduced LPS	Sulforaphan intervention increased the level of GLP1 in MASLD patients, which was positively correlated with the reduction in blood glucose and HOMA-IR
Camu-Camu	Agrinier et al. [51]	RCT	30 individuals with MASLD	Camu-camu for 12 weeks	- Reduced liver fat - Reduced AST, ALT - Increased in positive species of microbiota - Decreased in negative species of microbiota	No effect on body weight or adiposity
Inulin-propionate ester	Chambers et al. [143]	RCT	18 adults with MASLD	inulin-propionate ester for 42 days	Improvements in insulin sensitivity	
ITFs—inulin-type fructans	Reshef et al. [144]	RCT	19 adults with MASLD	ITFs vs. maltodextrin	- Liver fat content, fibroblast growth factor-19, and lipopolysaccharide-binding protein not improved - Body weight remained stable	
Metronidazole + inulin vs. placebo + inulin vs. placebo + placebo.	Lin Chong et al. [145]	RCT	60 participants with MASLD—biopsy proven	metronidazole + inulin 4 g twice daily vs. placebo twice daily + inulin 4 g twice daily vs. placebo + placebo	After VLCD: - Reduced BMI - Reduced ALT After metronidazole-inulin: - Reduced ALT	Metronidazole + inulin reduce ALT beyond that achieved after VLCD
Low glycemic index Mediterranean diet + physical activity	Calabrese et al. [146]	RCT	109 partecipants with MASLD	Low Glycemic Index Mediterranean Diet (LGIMD) vs. aerobic activity program (ATFIS_1) vs. combined activity program (ATFIS_2) vs. LGIMD + ATFIS_1 vs. ATFIS2 + Control Diet based on CREA-AN	Low glycemic index Mediterranean diet+ physical activity: - Reduced CAP - Increased in Firmicutes phylum - Increased in Ruminococcaceae	Lifestyle modifications, including diet and physical activity, affect the composition of gut microbiota in MASLD patients
A diet enriched in 1000 kcal/day	Jian et al. [147]	RCT	3 participants with overweight or obesity	A diet enriched in 1000 kcal/day of saturated fat/unsaturated fat/ simple sugars for 3 weeks	- Overfeeding saturated fat increased Proteobacteria - Unsaturated fat increased butyrate producers - Sugar overfeeding increased Lactococcus and E. Coli	The carriage of Bilophila was identified as a potential novel risk factor for diet-induced liver steatosis in humans
Fish vs. Fish + meat	He et al. [148]	RCT	34 patients with MASLD	Freshwater fish-based diet vs. freshwater fish-based + red meat-based diet for 84 days	In the fish group: - Reduction in hepatic steatosis - Improvement in ALT and GGT - Enrichment of Faecalibacterium, short-chain fatty acids, unconjugated bile acids - Depletion of Prevotella 9 and conjugated bile acids	Diet based on freshwater fish and red meat consumption did not exacerbate MASLD
Yogurt	Chen et al. [149]	RCT	100 women living with obesity, MASLD, and metabolic syndrome	Milk/yogurt for 24 weeks	Yogurt: - Ameliorated d IR and liver fat improving lipid metabolism - Lowered inflammation, oxidative stress, and LPS - Changed the gut microbiota composition	
